# Diagnostic value of thyroid-related serological indicators and pan-immune-inflammation value for differentiated thyroid carcinoma

**DOI:** 10.3389/fimmu.2025.1662638

**Published:** 2025-08-20

**Authors:** Yicheng Wen, Xin Yi, Jixin Shi, Peng Wan, Huan Wang, Li Tian, Huixia Liu

**Affiliations:** ^1^ School of Clinical Medicine, Chengdu University of Traditional Chinese Medicine, Chengdu, China; ^2^ School of Medicine, Tarim University, Alaer, China

**Keywords:** immune-inflammatory biomarkers, pan-immune-inflammation value, diagnostic value, thyroid-related serological indicators, differentiated thyroid carcinoma

## Abstract

**Background:**

Over the past decade, the remarkable rise in differentiated thyroid carcinoma (DTC) incidence, combined with the limitations of conventional diagnostic approaches, have prompted this study to explore the diagnostic value of thyroid-related serological indicators and pan-immune-inflammation value (PIV) for DTC, based on advancements in molecular biology and immunology.

**Methods:**

Based on postoperative pathological diagnosis, the present retrospective research comprised 112 individuals afflicted with DTC (observation group) and 93 individuals having benign thyroid tumors (control group) from January 2023 to January 2025. Differences in clinical data between the two groups were analyzed via univariate statistical methods. Logistic regression analyses identified influencing factors, while diagnostic value of thyroid-related serological indicators and PIV levels were evaluated through receiver operating characteristic analysis.

**Results:**

Intergroup differences regarding the levels of thyroxine (T4), free thyroxine (FT4), thyroid-stimulating hormone (TSH), thyroglobulin (Tg), lymphocyte count, monocyte count, and PIV were found to be significant (*p*<0.05). Additionally, negative correlation of lymphocyte count with DTC was noted by univariate binary logistic regression (odds ratio [OR] = 0.243, 95% confidence interval [CI]: 0.143–0.411]). TSH (OR=2.458, 95% CI: 1.690–3.575), FT4 (OR=1.383, 95% CI: 1.205–1.588), Tg (OR=1.008, 95% CI: 1.001–1.015), and PIV (OR=1.003, 95% CI: 1.000–1.005) were identified as independent influencing factors for DTC, and the area under the curve for their combination was 0.860 (95% CI: 0.809–0.912, sensitivity: 86.2%, specificity: 77.2%).

**Conclusion:**

This retrospective study suggested that TSH, FT4, Tg, and PIV were positively correlated with DTC, and their combination yielded the best diagnostic performance. It highlighted the potential utility of PIV as a novel immune-inflammatory biomarker and provided support for the development of DTC diagnosis.

## 1 Introduction

Thyroid carcinoma (TC), as the foremost pervasive endocrine cancer, is histologically categorized under three major types: differentiated, undifferentiated, and medullary, with differentiated thyroid carcinoma (DTC) constituting more than 90% of all cases (1, 2). There are two major histological DTC subtypes, namely papillary thyroid carcinoma, representing 81–90% of cases, and follicular thyroid carcinoma, comprising 4–11% of cases (3).

Despite the significantly heightened prevalence of DTC over the past decade, it has become one of the least lethal human cancers, since patients can usually be treated with thyroid hormone therapy, radioactive iodine therapy, surgery, and thyroid-stimulating hormone (TSH) therapy, which represents merely 0.3% of all cancer-related fatalities (4, 5). While the prognosis is favorable for most DTC patients, with long-term survival rates surpassing 90%, they face a notably high postoperative recurrence ranging from 25% to 35% (6, 7). Meanwhile, approximately 10% of DTC patients with a poor prognosis will develop lifelong locally advanced or widespread metastatic disease, which severely affects their quality of life (8). Hence, accurate and early diagnosis of DTC, along with the selection of appropriate therapeutic regimen, are key to further improving patient prognosis and elevating survival rates. Diagnosis of DTC is usually based on neck ultrasound and imaging examinations (CT, MRI, SPECT-CT and PET-CT) (2). However, due to the absence of distinct characteristics during the initial phase of DTC onset, its detection by the above traditional techniques is difficult to achieve, which can lead to problems such as misdiagnosis and missed diagnosis. Pathological examination is regarded as the “gold standard” for DTC. Nevertheless, the risk of trauma associated with this procedure can compromise patient tolerance for the diagnosis (9). Currently, with the advancements in molecular biology and immunology, the measurement of some thyroid-related serological indicators, such as TSH and thyroglobulin (Tg) levels, has emerged as a promising diagnostic method for DTC (10). Similarly, a new immune-inflammatory biomarker called pan-immune-inflammation value (PIV) has been postulated and validated as a great predictor in various malignant tumors (11). PIV integrates counts of neutrophils, monocytes, platelets, and lymphocytes, which are the key mediators of cancer-related inflammation and can reflect the inflammatory and immune status of tumors (12). Meanwhile, in contrast to individual blood cell parameters, PIV may provide a more comprehensive reflection of the complex characteristics of systemic immune and inflammatory states. In DTC, elevated PIV may indicate alterations in the tumor microenvironment. This study further explored the diagnostic value of thyroid-related serological indices and PIV for DTC, aiming to compare the diagnostic efficacy of individual and combined detection of various indicators, establish a better diagnostic model, and provide a more accurate evidence-based reference for the clinical diagnosis of DTC.

## 2 Methods

### 2.1 Patients selection

**Figure 1:**
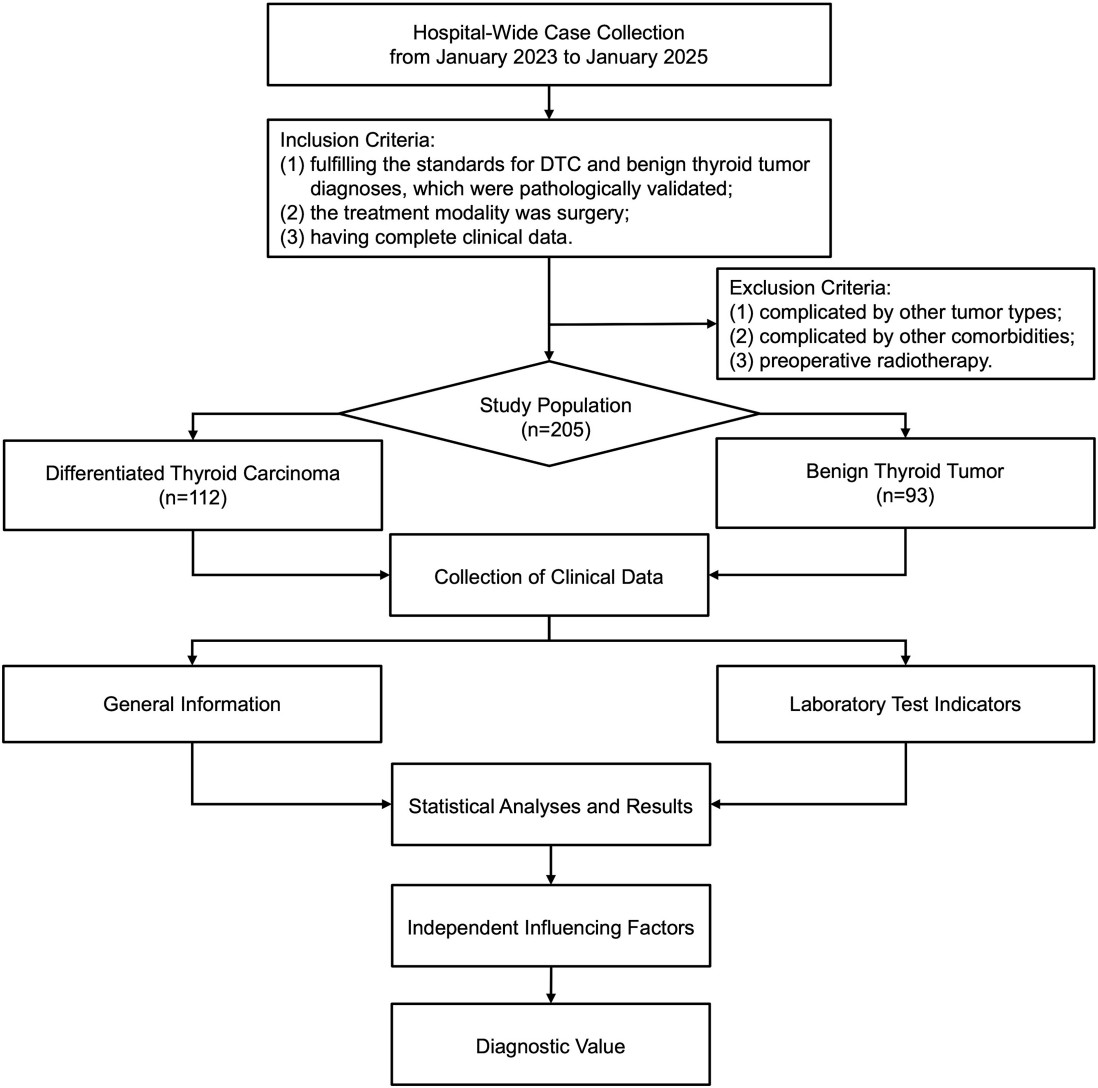
Flow diagram for the study.

A retrospective analysis was conducted (**Figure 1**), which enrolled 112 patients with DTC treated at the Hospital of Chengdu University of Traditional Chinese Medicine from January 2023 to January 2025. The observation group comprised 31 males and 81 females, who were aged between 25 and 69 years. A control group was constituted by selecting 93 patients (22 males and 71 females) diagnosed with benign thyroid tumors during the same period at the hospital, who were aged between 21 and 75 years. Inclusion criteria: (1) fulfilling the standards for DTC and benign thyroid tumor diagnoses, which were pathologically validated (postoperative pathological examinations) (13); (2) the treatment modality was surgery; (3) having complete clinical data (general information, preoperative laboratory report, postoperative pathology report). Exclusion criteria: (1) complicated by other tumor types; (2) complicated by infections or severe cardiac, hepatic, renal insufficiency and other comorbidities; (3) preoperative radiotherapy. Ethical approval was gained for the present research from the Ethics Committee of the aforementioned hospital, while informed consent was waived given its retrospective nature (approval no. 2025KL-036).

### 2.2 Collection of clinical data

Clinical data were collected after patient admission, including: (1) General information: age, gender; (2) Laboratory test indicators: TSH, Tg, triiodothyronine (T3), thyroxine (T4), free triiodothyronine (FT3), free thyroxine (FT4), parathyroid hormone (PTH), as well as neutrophil, lymphocyte, monocyte, and platelet counts.

All laboratory indicators were tested within a two-week period before surgery. Fasting venous blood specimens were obtained from all participants in the morning and dispensed into ethylenediaminetetraacetic acid (EDTA) anticoagulant tubes and plain serum tubes. For serum-based indicator assays, samples in serum tubes were allowed to stand at room temperature for 30 minutes, then centrifuged at 4500 rpm for 10 minutes. The separated serum was analyzed by automated chemiluminescence immunoassay analyzer (Roche, Germany) with related reagents to quantify TSH, Tg, T3, T4, FT3, FT4, and PTH. For complete blood count, after confirming no macroscopic coagulation in EDTA-anticoagulated samples, and platelet, neutrophil, monocyte, and lymphocyte counts (×10^9/L) were measured using an automated hematology analyzer (Abbott, USA).

### 2.3 Calculation method of PIV

The following computational formula for PIV, as an indicator of systemic inflammation status and a non-invasive and valid potential prognostic indicator of patients suffering from diverse malignancies, was employed: PIV = (neutrophil count × monocyte count × platelet count)/lymphocyte count, with each count unit expressed as ×10^9/L (14).

### 2.4 Statistical analysis

Data statistics and analysis were accomplished using SPSS 27.0 software. For measurement data, the Shapiro-Wilk test was employed to examine normality. Data obeying normally distribution were reported as means ± standard deviations (SDs), with intergroup comparisons made via the independent samples t-test. In contrast, non-normal data were depicted by medians with interquartile ranges (IQR; 25th to 75th percentiles), and their intergroup comparisons utilized the Mann-Whitney U-test. For the assessment of categorical data, either Fisher’s exact or Chi-square test was employed. Correlations among indicators were analyzed by Spearman’s correlation analysis, and the subsequent heatmap plotting was achieved via “corrplot” function in R (ver. 4.4.3). Influencing factors were screened by conducting univariate and multivariate binary logistic regression analyses. The diagnostic value of thyroid-related serological indicators and PIV levels was evaluated by the receiver operating characteristic (ROC) analysis, and the areas under the curves (AUCs) were determined. *p* value< 0.05 was regarded as statistically significant.

## 3 Results

### 3.1 Intergroup comparison of clinical data

The intergroup comparative analysis on clinical data revealed absence of significant differences in general information (age, gender), suggesting that the clinical data of the two patient groups were comparable. By contrast, the intergroup differences regarding the levels of TSH, T4, FT4, Tg, lymphocyte count, monocyte count, and PIV were significant (*p*<0.05) (**Table 1**).

**Table 1:** Intergroup comparison of clinical data.

Indicators	Observation group (*n*=112)	Control group (*n*=93)	*Z/χ²*	*p* value
Age (year)^a^	42.00(33.75,52.25)	45.00(32.00,54.00)	-0.085	0.932
Gender^b^			0.429	0.513
Male	31(27.68)	22(23.66)		
Female	81(72.32)	71(76.34)		
TSH (ulU/mL) ^a^	2.40(1.64,3.41)	1.36(1.00,2.35)	-5.455	<0.001
T4 (nmol/L) ^a^	98.80(90.40,113.00)	86.32(69.66,108.00)	-3.063	0.002
T3 (nmol/L) ^a^	1.68(1.53,1.86)	1.81(1.34,2.19)	-1.246	0.213
FT4 (pmol/L) ^a^	16.50(15.20,18.50)	14.10(11.92,16.50)	-6.064	<0.001
FT3 (pmol/L) ^a^	4.72(4.42,5.12)	4.75(4.12,5.51)	-0.465	0.642
Tg (ng/mL) ^a^	57.30(20.10,91.00)	22.00(11.40,58.40)	-4.107	<0.001
PTH (pg/mL) ^a^	56.02(39.40,74.63)	58.43(43.20,81.58)	-1.174	0.240
NEUT (10^9/L) ^a^	3.71(2.88,4.70)	3.60(2.91,4.69)	-0.257	0.797
LYMPH (10^9/L) ^a^	1.43(1.16,1.73)	2.08(1.37,2.63)	-5.149	<0.001
MONO (10^9/L) ^a^	0.38(0.26,0.58)	0.27(0.21,0.42)	-3.246	0.001
PLT (10^9/L) ^a^	224.00(194.00,255.00)	223.00(182.00,261.00)	-0.058	0.954
PIV ^a^	206.54(134.21,309.83)	119.60(78.11,233.28)	-5.049	<0.001

TSH, thyroid-stimulating hormone; T4, thyroxine; T3, triiodothyronine; FT4, free thyroxine; FT3, free triiodothyronine; Tg, thyroglobulin; PTH, parathyroid hormone; NEUT, neutrophil count; LYMPH, lymphocyte count; MONO, monocyte count; PLT, platelet count; PIV, pan-immune-inflammation value.

a Median (interquartile range).

b n (%).

### 3.2 Results of univariate binary logistic regression

Taking tumor group as the dependent variable (1 = observation group, 0 = control group), we performed univariate binary logistic regression analyses for each of the significantly different indicators (TSH, T4, FT4, Tg, lymphocyte count, monocyte count, PIV), with a view to identifying the significant influencing factors for DTC. According to our results, TSH, T4, FT4, Tg, lymphocyte count, monocyte count, and PIV exhibited statistical significance (*p*<0.05), and the lymphocyte count was linked negatively to DTC (odds ratio [OR] = 0.243, 95% confidence interval [CI]: 0.143–0.411]) (**Table 2**).

**Table 2:** Univariate binary logistic regression results.

Indicators	OR (95% CI)	SE	*p* value
TSH (ulU/mL)	2.187(1.607-2975)	0.157	<0.001
T4 (nmol/L)	1.020(1.007-1.033)	0.006	0.002
FT4 (pmol/L)	1.401(1.247-1.575)	0.060	<0.001
Tg (ng/mL)	1.012(1.005-1.019)	0.004	0.001
LYMPH (10^9/L)	0.243(0.143-0.411)	0.369	<0.001
MONO (10^9/L)	8.567(1.752-41.893)	0.809	0.008
PIV	1.004(1.002-1.006)	0.001	0.001

SE, standard error; OR, odds ratio; CI, confidence interval.

### 3.3 Correlations among indicators

**Figure 2:**
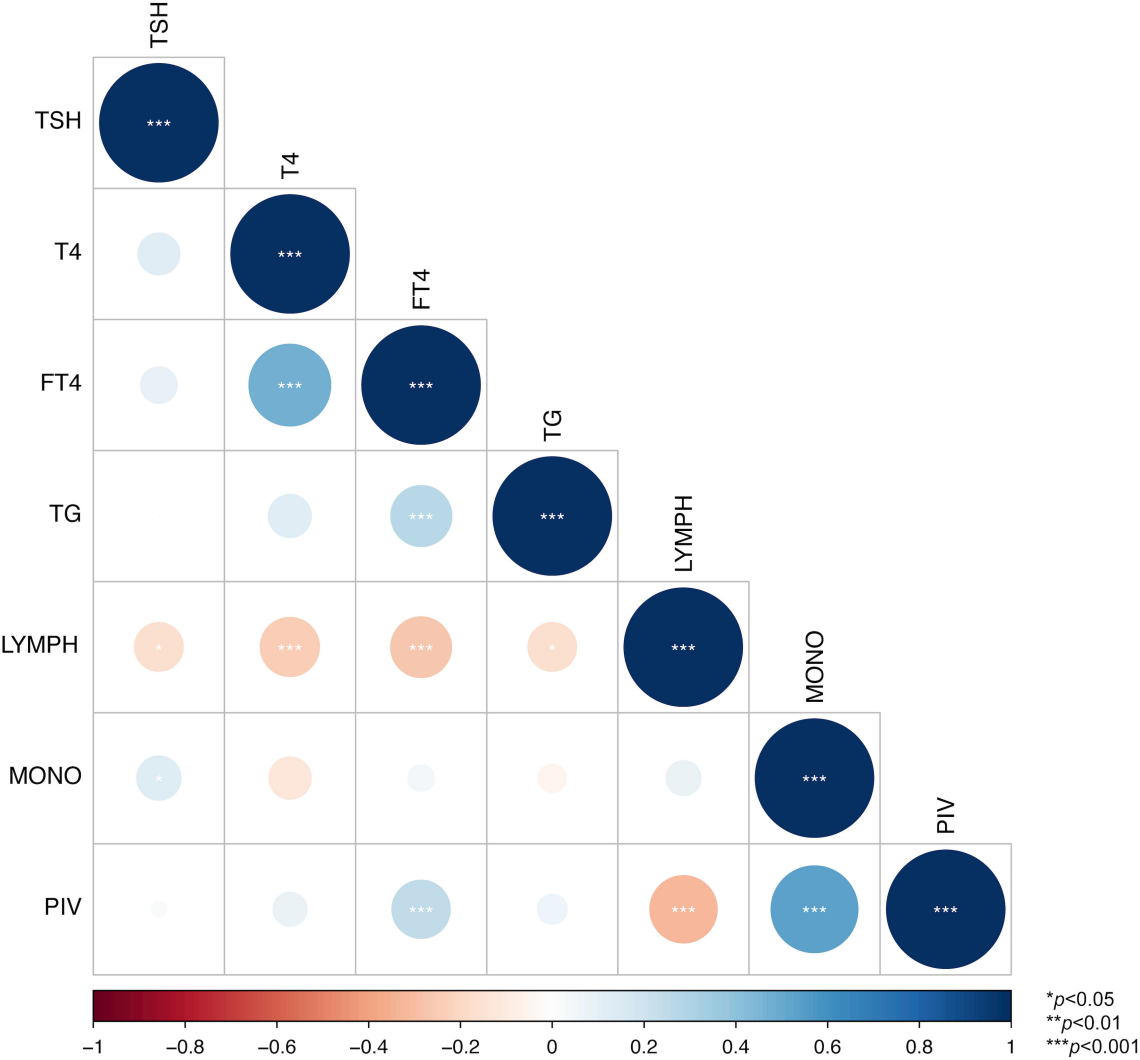
Correlation heatmap among indicators. * indicates p < 0.05, ** indicates p < 0.01, and *** indicates p < 0.001.

Spearman correlation analyses were performed among the significant indicators (TSH, T4, FT4, Tg, lymphocyte count, monocyte count, PIV) identified from the above univariate regression analyses. Our results demonstrated that both the lymphocyte count (r=-0.32) and monocyte count (r=0.54) exhibited moderate to strong and statistically significant correlations with PIV (*p* < 0.001). Given that these two cell counts are components included in the PIV calculation, they were not considered as separate predictive diagnostic indicators. Additionally, T4 and FT4 showed a moderate to strong and significant correlation (r=0.47, *p* < 0.001).Since FT4 more directly reflects thyroid function and is not influenced by protein binding in most clinical diagnostic scenarios, T4 was excluded as a predictive diagnostic indicator (15) (**Figure 2**).

### 3.4 Results of multivariate binary logistic regression

Taking tumor group as the dependent variable (1 = observation group, 0 = control group), and TSH, FT4, Tg, and PIV as independent variables, we conducted multivariate binary logistic regression analyses. According to our results, TSH (OR=2.458, 95% CI: 1.690–3.575), FT4 (OR=1.383, 95% CI: 1.205–1.588), Tg (OR=1.008, 95% CI: 1.001–1.015), and PIV (OR=1.003, 95% CI: 1.000–1.005) were independent influencing factors for DTC (*p*<0.05) (**Table 3**).

**Table 3:** Multivariate binary logistic regression results.

Indicators	OR (95% CI)	SE	*p* value
TSH (ulU/mL)	2.458(1.690,3.575)	0.470	<0.001
FT4 (pmol/L)	1.383(1.205,1.588)	0.097	<0.001
Tg (ng/mL)	1.008(1.001,1.015)	0.036	0.020
PIV	1.003(1.000,1.005)	0.001	0.029

### 3.5 Diagnostic efficacy of various indicators

**Table 4**; **Figure 3** display the diagnostic efficacy results for various indicators, along with their combination, in the ROC curve analysis. Clearly, a combined detection of TSH, FT4, Tg, and PIV achieved the highest diagnostic efficacy for DTC (AUC=0.860, 95% CI: 0.809–0.912), with respective sensitivity and specificity levels of 86.2% and 77.2%. Among single indicators, FT4 exhibited the highest diagnostic value for DTC (AUC=0.744, 95% CI: 0.674–0.813), showing an 89.9% sensitivity and a 50.0% specificity. The AUC for TSH was 0.733 (95% CI: 0.655–0.802), with an 85.3% sensitivity alongside a 54.3% specificity. Tg (AUC=0.671, 95% CI: 0.597–0.744) had lower sensitivity (33.9%) but high specificity (95.7%). Additionally, the AUC for PIV was 0.706 (95% CI: 0.633–0.779), showing a 73.4% sensitivity alongside a 64.1% specificity. The combined detection and each individual indicator had *p* values < 0.001, indicating statistically significant results.

**Table 4:** Diagnostic efficacy of various indicators in patients with DTC.

Indicators	AUC (95% CI)	Sensitivity (%)	Specificity (%)	Youden Index	Cut-off Value	*p* value
TSH	0.733(0.655-0.802)	85.3	54.3	0.396	1.39	<0.001
FT4	0.744(0.674-0.813)	89.9	50.0	0.399	13.70	<0.001
Tg	0.671(0.597-0.744)	33.9	95.7	0.296	81.10	<0.001
PIV	0.706(0.633-0.779)	73.4	64.1	0.375	137.51	<0.001
Combined detection	0.860(0.809-0.912)	86.2	77.2	0.634	–	<0.001

AUC, area under the curve.

**Figure 3:**
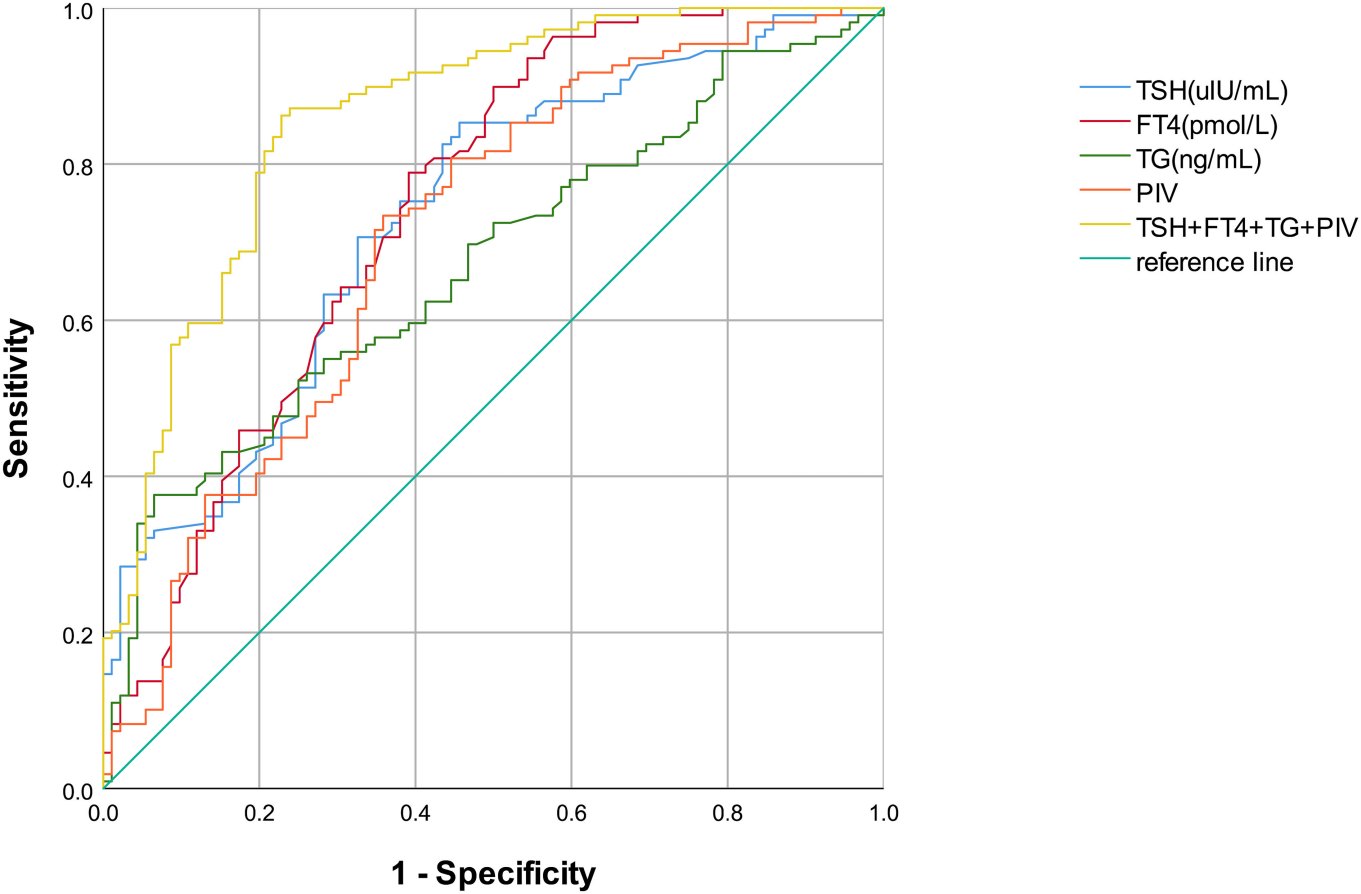
ROC curves for DTC diagnosis using thyroid-related serological indicators and PIV.

## 4 Discussion

Benign thyroid tumors and DTC frequently exhibit overlapping clinical manifestations and physical findings (16). While DTC typically displays a positive prognosis, its indolent biological and clinical course frequently leads to metastasis to cervical lymph nodes, either at the initial diagnosis or during the postoperative surveillance (17). Consequently, accurate preoperative assessment and regular postoperative monitoring are essential for optimal disease management (18).

As far as we are aware, our research represents the first attempt to systematically explore the diagnostic value of PIV and its combination with thyroid-related serological indicators for DTC. We found that TSH, FT4, Tg, and PIV were independent influencing factors for DTC, and their combined detection yielded the highest diagnostic value, which may reduce misdiagnosis and enhance clinical diagnostic accuracy.

A previous meta-analysis, which examined the association of TSH with TC risk, also demonstrated significantly elevated risks of DTC with increasing TSH concentrations (19). The outcomes of the present study support these findings, and further investigation showed that TSH had preferable diagnostic value for DTC. This may be attributable to the potential contribution of elevated TSH levels to the growth of thyroid epithelial cells. TSH can induce the release of pro-inflammatory cytokines such as IL-6 and TNF-α in thyroid follicular cells through multiple receptor signaling pathways (e.g., cAMP/PKA, NF-κB), thereby establishing a pro-inflammatory microenvironment (20, 21). This process in turn instigates repeated inflammatory reactions in the gland and triggers the body’s autoimmune mechanisms, thus placing the thyroid gland in a long-term state of autoimmune abnormality, ultimately resulting in the abnormal growth of thyroid tissues and the formation of tumors (22). Nevertheless, the correlations between FT4, FT3 levels and the probability of developing DTC are still a matter of debate. One study involving a population whose thyroid hormone levels were normal demonstrated that higher FT4 levels were linked positively to the TC risk (23). In a study assessing the association of thyroid function with DTC, it was found that even though patients had normal FT3 and FT4 levels, lower concentrations of these hormones were linked to a heightened risk of developing TC, irrespective of the patient gender or the type of thyroid nodule (24). According to a meta-analysis, FT3 levels were negatively linked to the TC risk, while FT4 levels showed no correlation with TC (25). These disparities may arise not only from population heterogeneity, such as geographic variations in iodine nutrition and genetic polymorphisms in thyroid-related genes, but also from methodological variations, such as differences in immunoassay platforms and inconsistent laboratory reference intervals (26). Notably, our findings partially align with the first-mentioned association regarding FT4’s diagnostic value, in which FT4 elevations correlated positively with DTC. FT4 was an independent DTC predictor with the best diagnostic performance among thyroid-related serological indicators. Suggestively, elevated FT4 levels indicate the presence of circulating TSH receptor-stimulating antibodies (TSHR-Abs), which activate identical intracellular signaling pathways to TSH, and lead to enhanced proliferation and attenuated apoptosis of thyroid follicular cells, thereby facilitating tumorigenesis (27).

Tg, synthesized by thyroid follicular cells, acts as a critical precursor for the production of thyroid hormones; it is also synthesized by well-differentiated malignant thyroid tissue and functions as one of the major antigens in autoimmune thyroid diseases (28). According to a few recent reports, the diagnosis and metastasis of DTC are significantly influenced by preoperative Tg levels (29, 30). Moreover, a large sample-size cohort study within the European Prospective Investigation into Cancer and Nutrition (EPIC) has shown a strong positive association between TC risk and pre-diagnostic blood Tg (31). The conclusions of the above studies agree with the findings of ours. However, in our multivariate logistic regression analysis, the OR for Tg was 1.008 (95% CI: 1.001–1.015). Although this is statistically significant, the effect size is small, indicating that each 1 ng/mL increase in serum Tg is associated with only an 0.8% rise in the odds of DTC. Such a small effect size may reflect the influence of multiple factors on Tg levels (e.g., anti-Tg antibodies) and suggests that early or minute DTC lesions produce only limited changes in serum Tg (32). Although the effect size of Tg is small, its clinical significance may lie in its cumulative effect: for example, a 50 ng/mL increase in Tg corresponds to a greater than 40% elevation in the odds of DTC. Furthermore, ROC analysis demonstrated that Tg retains exceptionally high specificity (95.7%). This makes it particularly useful for confirming DTC in patients with indeterminate ultrasound or cytology findings and facilitates risk stratification when combined with other biomarkers. Additionally, Tg’s sensitivity was only 33.9%, indicating a considerably high risk of missed diagnosis if used alone as a screening tool, despite its excellent performance in ruling out false positives. This limitation suggests that Tg is more suitable as an auxiliary indicator and should be used in conjunction with other biomarkers with higher sensitivity to enhance overall diagnostic efficiency and reduce the rate of missed diagnoses.

Immuno-inflammatory biomarkers, including neutrophils, lymphocytes, monocytes, and platelets, are essential for evaluating the equilibrium between immune and inflammatory states in patients. Numerous studies have investigated their value as diagnostic and prognostic biomarkers for cancer, finding that they possess significant diagnostic and outcome predictive value (33). In recent years, PIV, a novel parameter for prognostic evaluation developed based on the aforementioned four immuno-inflammatory biomarkers, has demonstrated significant prognostic value across various malignancies, including prostate cancer (34), esophageal squamous cell carcinoma (35), metastatic colorectal cancer (36), and pulmonary carcinoma (37). However, to our knowledge, its diagnostic value for and correlation with DTC have never been documented in the existing literature. Hence, by incorporating the aforementioned indicators, the present study analyzed their associations with DTC, as well as the diagnostic value of PIV. We discovered a positive correlation of PIV elevation with DTC, and the role of PIV as an independent predictor for DTC with good diagnostic and predictive performance. Prior studies have shown that platelets can activate epithelial–mesenchymal transition in tumor cells, while neutrophils facilitate tumor growth and metastasis through cytokine secretion (38, 39). However, our study revealed no significant disparities in the platelet or neutrophil count between DTC patients and those having benign thyroid tumors, while observing significant differences in the monocyte and lymphocyte counts. Furthermore, in univariate binary logistic regression analyses, we noted a significant positive association between monocyte count and DTC, whereas a significant negative association between lymphocyte count and DTC. This may be related to the mechanisms by which monocytes promotes tumor cell invasion, metastasis, and angiogenesis, i.e. monocytes is recruited into the tumor microenvironment, where they become activated and function as tumor-associated macrophages, secreting a variety of cytokines (40, 41). Lymphocytes, on the other hand, may suppress the initiation and progression of DTC by mediating antitumor immune responses, such as T cell-mediated cytotoxicity and immune surveillance (42, 43). Therefore, composite biomarkers like PIV, which incorporate multiple peripheral immune cell subsets, may reflect systemic immune activation and characterize the intricate tumour-host interactions (44). From an immunological perspective, elevated PIV levels may more directly reflect the imbalance between pro-tumor inflammation and anti-tumor immunity in the tumor microenvironment (45, 46). This not only provides theoretical support for in-depth analysis of the immunological mechanisms linking inflammation and DTC, but also offers a clear basis for clarifying its potential value in DTC diagnosis. Although PIV showed a strong correlation with lymphocyte and monocyte counts in Spearman correlation analysis, the intrinsic association between these components does not diminish the independent predictive value of PIV as a composite immune-inflammatory biomarker. To avoid the adverse impact of multicollinearity on the model, we excluded lymphocyte and monocyte counts from the multivariate logistic regression. Even so, PIV still exhibited a significant independent association with DTC in the multivariate logistic regression analysis. This result suggests that by integrating the prognostic information of different immune-inflammatory cells, PIV can provide favorable diagnostic value for DTC, which is consistent with previous studies in the field of oncology (34–37).

These findings provide a theoretical basis for optimizing noninvasive preoperative DTC diagnosis while demonstrating clinical potential to address pathological diagnostic limitations. However, this study has several limitations. First, its retrospective design introduces inherent constraints, including potential selection bias in patient enrollment, reliance on the completeness and accuracy of existing medical records and laboratory data, and the inability to control for unmeasured confounders. Second, since the research was carried out at a single institution and was constrained by departmental limitations, the initial lack of sample diversity could potentially introduce bias into the results. Third, the absence of healthy controls restricts direct assessment of biomarker performance in cancer screening. While benign tumor patients represent the clinically relevant differential diagnosis group, this design may underestimate diagnostic specificity. Consequently, the reliability of these findings requires validation through additional multicenter studies. Specifically, future prospective research involving diverse populations (e.g., varying geography, ethnicity, and age) is needed to validate and generalize the diagnostic findings for PIV and serological biomarkers in DTC.

## 5 Conclusion

This study has clarified to some extent the correlations of thyroid-related serological indicators and PIV with DTC and has tentatively established the diagnostic value of relevant indicators. TSH, FT4, Tg, and PIV were positively correlated with DTC, and their combination yielded the best diagnostic performance. Meanwhile, considering the low cost and high accessibility of routine blood and thyroid function tests, this approach demonstrates particular suitability for initial screening in resource-limited areas—effectively reducing the rate of missed diagnoses of DTC. Consequently, future research should entail large-scale, prospective, multicenter cohort studies aimed at more robustly validating the diagnostic accuracy of thyroid-related serological indicators combined with PIV. These findings hold the potential to improve early screening, risk stratification, and dynamic monitoring of DTC, providing support for the development of DTC diagnosis and treatment.

## Data Availability

The raw data supporting the conclusions of this article will be made available by the authors, without undue reservation.
